# Changes in social connection during COVID-19 social distancing: It’s not (household) size that matters, it’s who you’re with

**DOI:** 10.1371/journal.pone.0245009

**Published:** 2021-01-20

**Authors:** Karynna Okabe-Miyamoto, Dunigan Folk, Sonja Lyubomirsky, Elizabeth W. Dunn

**Affiliations:** 1 Department of Psychology, University of California, Riverside, Riverside, California, United States of America; 2 Department of Psychology, University of British Columbia, Vancouver, B. C., Canada; University of Pennsylvania, UNITED STATES

## Abstract

To slow the transmission of COVID-19, countries around the world have implemented social distancing and stay-at-home policies—potentially leading people to rely more on household members for their sense of closeness and belonging. To understand the conditions under which people felt the most connected, we examined whether changes in overall feelings of social connection varied by household size and composition. In two pre-registered studies, undergraduates in Canada (N_Study 1_ = 548) and adults primarily from the U.S. and U.K. (N_Study 2_ = 336) reported their perceived social connection once before and once during the pandemic. In both studies, living with a partner robustly and uniquely buffered shifts in social connection during the first phases of the pandemic (β_Study 1_ = .22, β_Study 2_ = .16). In contrast, neither household size nor other aspects of household composition predicted changes in connection. We discuss implications for future social distancing policies that aim to balance physical health with psychological health.

## Introduction

On March 11, 2020, the Centers for Disease Control and Prevention [1] declared the COVID-19 outbreak a pandemic. By early April, COVID-19 had already spread to nearly 1.5 million people worldwide [2]. In an effort to slow down its transmission, countries around the world implemented social/physical distancing guidelines [3], compelling individuals to stay at least 6 feet (2 meters) away from anyone outside their household [4]. Early in its implementation, the WHO [2] announced that it would be moving away from the term “social distancing” and begin using “physical distancing” to more accurately describe the practice. However, the original term stuck, especially in the U.S., U.K., Australia, Italy, France, Poland, Russia, India, South Korea, and Hong Kong, even though the very label of “social distancing” arguably undermines feelings of social connection. We use “social distancing” in this paper to reflect common usage. Such non-pharmaceutical public health interventions have been long proposed to reduce the spread of infectious disease. For example, mathematical modeling suggests that social distancing can reduce transmission of influenza by over 90% [5], and retrospective analyses of past pandemics (e.g., in 1918–1919) show that areas that implemented social distancing measures earlier were slower to reach peak and total mortality rates [6]. However, although social distancing policies have historically helped protect physical health worldwide, these policies have also greatly limited people’s range of social interactions, an important cost to weigh against their benefits.

Understanding the ways in which policy makers can balance physical health and psychological health while continuing social distancing has generated recent interest [7–9]. This calculus is crucial, as social distancing for extended periods of time may strain people’s needs for social connection to such an extent that they may eventually disregard policy guidelines. Social connection, or a sense of belonging and closeness with others, is fundamental to human development and well-being [10–13]. For example, having frequent social interactions and spending more time talking with others are both associated with greater well-being [14–16]. Furthermore, experiments have shown that people prompted to engage in more social interactions relative to control activities report higher levels of positive emotion and social connectedness [17–19]. In sum, understanding the conditions under which social connection is maximized during COVID-19 social distancing may inform future policies that can strike a balance between ensuring that people continue to social distance to protect physical health and ensuring that they stay socially connected to protect psychological health.

Social distancing initiatives have led millions of people globally to stay in their homes [20], abruptly forcing individuals to rely on their household members for their sense of overall social connection. This shift may pose a risk for those living alone, who report experiencing relatively more loneliness even under normal circumstances [21, 22]. Living in a larger household has been shown to be protective of loneliness [23], suggesting that living in larger households may safeguard people from declines in social connection during the pandemic. In light of the stressful and uncertain nature of the pandemic, a larger household may offer not only more opportunity for social interactions but greater social support, which is associated with well-being [24]. However, living in bigger households, which requires sharing a space day in, day out with the same people, may lead to greater tension, conflict, and sense of being crowded [25].

Living with a partner in particular may offer unique advantages [21], especially during stressful times [26]. Living with a partner is also protective of loneliness compared to being single and living alone—and even compared to having a partner but not cohabiting [21]. In a large study following 30,000 people, the most important social behavior that predicted well-being was the amount of time spent with a partner [27]. However, although the weight of the evidence supports the benefits of living with partners on social connection, the stress caused by the pandemic—and the friction associated with couples forced to spend all day together in close quarters (see [28], for examples)—may also negatively impact relationships [29].

Aside from partners, other household members may also provide feelings of closeness and opportunities for interaction. For example, living with children is linked with higher well-being [30] and lower levels of loneliness [31], and so is sharing a household with pets [32]. However, such benefits may be limited during a pandemic in which children are homeschooled, parents are working remotely from home or else looking for work, and neither pet owners nor their pets are able to interact socially with their peers.

Unlike social distancing policies during past pandemics, COVID-19 is unique because people today have the ability to connect digitally not only by phone, but through the use of social media, video calling, and text messaging. However, although connecting via digital and social media has been found to enhance offline relationships [33, 34], digital communication often feels unnatural and lacks rich nonverbal cues, which may hinder mutual understanding [35] and be cognitively taxing [36]. In times of stress and crisis, these forms of online communication may in turn promote other negative outcomes, such as “Zoom fatigue” [37]. Thus, face-to-face interactions with household members are likely to be essential to increased feelings of social connectedness.

In sum, social connection is crucial for both psychological and physical health, perhaps especially so during an unprecedented global pandemic that has claimed more lives than every war since the Korean War [38]. How can future policy guidelines balance protecting physical health through social distancing with protecting psychological health by maintaining feelings of connection? To understand the conditions under which people felt the most connected, we examined whether changes in overall feelings of social connection varied as a function of household size and composition.

## Present research

In two pre-registered studies of undergraduates at a Canadian university (N_Study 1_ = 548) and adults primarily residing in the United States and United Kingdom (N_Study 2_ = 336), we followed individuals before and during the COVID-19 pandemic to examine changes in feelings of social connection based on (1) household size and (2) household composition. Using two-tailed tests, we tested the following primary hypotheses. First, we expected that people in larger households to show relatively smaller declines (or bigger increases) in social connection as a result of the COVID-19 pandemic. Second, we hypothesized that household composition would predict changes in social connection as a result of the COVID-19 pandemic. In examining household composition, we focused on whether participants lived with a partner (or not), lived with a pet (or not), and were caregivers (or not). Feelings of social connection were assessed with three different measures—the Social Connectedness Scale (Study 1; [39]), the relatedness subscale of the Balanced Measure of Psychological Needs (BMPN; Study 2; [40]), and the UCLA Loneliness Scale (Study 2; [41]).

## Study 1

Undergraduates at the University of British Columbia completed our measures as part of two separate surveys. We obtained ethics approval from the Behavioral Research Ethics Board at the University of British Columbia, and participants provided written consent to be part of our study. The first survey was completed before the COVID-19 pandemic (Time_1_), and the second survey was completed during the COVID-19 pandemic (Time_2_). We pre-registered our analysis plan and stopping rules on the OSF and they are available at [https://tinyurl.com/yddwt28v]. A separate pre-registered study that used a portion of the data to answer a different research question can also be found on the OSF at [https://tinyurl.com/ybwz8ufb].

## Method

### Time_1_

Between January 6, 2020 and the end of March 2020, 3,504 participants completed demographic questions and a social connection measure alongside other items as part of an optional department-wide pre-screening. For consistency with Study 2, we only included participants who completed this questionnaire on or before February 12, 2020, resulting in a Time_1_ sample of 2,903 students. After removing participants who were missing more than two items on the social connection measure (as pre-registered), we obtained a total sample of 2,708 eligible participants.

### Time_2_

We invited participants who had completed pre-screening at Time_1_ to complete a second survey between April 1–8^th^, 2020. The Time_2_ survey consisted of the same measure of social connection as in the Time_1_ survey, as well as measures assessing students’ living arrangements, behaviors, and experiences during the COVID-19 pandemic. A total of 1,059 participants completed the Time_2_ survey. As pre-registered, 8 participants were removed for responding 12 or more times in a row with the same answer on the social connectedness measure and 1 participant was removed for failing to answer more than 2 items on the social connectedness measure. Although not pre-registered, we also removed participants who did not supply an ID number to link responses (*n* = 125) or completed the survey twice (*n* = 22), For those who completed the survey twice, we only included their responses from the first survey.

Of the remaining 903 participants, 548 participants (*M*_*age*_ = 20.78, *SD*_*age*_ = 2.96; 77% women) completed both surveys and met our inclusion criteria. Participants in this final dataset did not significantly differ from the remaining eligible participants who completed the Time_1_ survey in Time_1_ social connection (*p* = .359) or household income (*p* = .154). Because we aimed to recruit as many participants as possible, we did not conduct an a priori power analysis; however, based on sensitivity analyses using GPower [42] and assuming two-tailed α = 0.05 and 80% power, we should have been able to detect a small effect size of ƒ^2^ = .01 (R_adj_^2^ = .02) in a 2-predictor regression model and ƒ^2^ = .01 (R_adj_^2^ = .001) in a 5-predictor regression model. R_adj_^2^ is reported in the manuscript. The dataset for the final sample can be found on the OSF at [https://tinyurl.com/y7nvg5vf].

## Measures

The measures for Study 1 can be found on OSF at [https://tinyurl.com/y7jfk4al].

### Social connection

Social connection was assessed with the revised 20-item Social Connectedness Scale [39]. Participants indicated their level of agreement with items such as, “I feel close to people” and “I feel understood by the people I know” (1 = *strongly disagree*, 6 = *strongly agree*). We removed the item, “I feel comfortable in the presence of strangers” from both time points, because it may have had a different meaning in the midst of the pandemic. Participants completed the measure at Time_1_ with reference to their general view of themselves (*α =* .94). At Time_2_ however, due to the rapid changes to daily life that participants were experiencing, we asked them to think about the past week (*α* = .93).

### Household size and composition

To assess household size, we asked participants “other than yourself, how many people are currently living in the same place you are now?” with answer choices ranging from “living alone” to “10+ people.” For each person in their household, participants specified whether the person was a “spouse/partner/girlfriend/boyfriend” (subsequently referred to as *partner*), “child,” “parent,” “brother/sister,” “other family member,” “friend,” “roommate/acquaintance,” “live-in help,” or “other.” Participants could only select one option per household member.

### Living with pets

We asked whether participants were “currently living with any pets” (yes/no).

### Being a caregiver

Participants were asked whether they were “currently the primary caregiver for anyone else (e.g., children or elderly family members)” (yes/no).

### Social/physical distancing

Participants indicated whether they were “currently practicing social or physical distancing,” and to indicate how many people aside from their household members got to within 6 feet or less of them on the previous day.

### Hours spent video calling with family and friends

Participants were asked “yesterday, how many hours did you video call with family and friends” with answer choices ranging from “0” to “10+ hours.”

### Study 1 results

The code used to conduct the Study 1 analyses can be found on the OSF at [https://tinyurl.com/y7b8cnw3]. Correlations between all variables in Study 1 can be found in Table 1.

**Table 1:** Correlations among variables (Study 1).

	Household Size	Living Alone	Living with Partner	Living with Child(ren)	Living with Pet	Being a Caregiver	Hours Video Calling	Social Distancing	T1 Connectedness	T2 Connectedness
Household Size	1									
Living Alone	-.50***	1								
Living with Partner	-.06	-.12**	1							
Living with Child(ren)	.05	-.03	.10*	1						
Living with Pet	.19***	-.17***	.04	.03	1					
Being a Caregiver	.08	-.03	.07	.40***	.07	1				
Hours Video Calling	-.01	.02	-.07	.10*	-.02	.05	1			
Social Distancing	.05	-.10*	.05	.01	-.02	.01	.03	1		
T1 Connectedness	.06	-.08	-.01	-.01	.05	.03	.12**	.04	1	
T2 Connectedness	.06	-.09*	.08	-.03	-.03	.03	.14***	.04	.64***	1

Note.

*** = p < .001

** = p < .01.

* = p < .05.

#### Did household size buffer changes in social connection as a result of the COVID-19 pandemic?

As reported in Folk et al. [43], our sample exhibited a slight but significant decrease in feelings of social connectedness from Time_1_ to Time_2_, and 98% of participants indicated they were social/physical distancing (see Table 2).

**Table 2:** Means and standard deviations for household size and composition (Study 1).

Household Variable	Sample Size	Time 1 Connectedness	Time 2 Connectedness	Social Distancing	Six Feet
Full Sample	548	4.11 (0.85)	3.98 (0.83)	98% Yes	0.74 (1.35)
Living Alone	49	3.90 (0.95)	3.74 (0.82)	94% Yes	0.67 (1.18)
Not Living Alone	499	4.13 (0.84)	4.00 (0.83)	99% Yes	0.74 (1.37)
Living with Partner	67	4.10 (0.98)	4.16 (0.89)	100% Yes	0.91 (1.58)
Not Living with Partner	481	4.12 (0.84)	3.96 (0.82)	98% Yes	0.71 (1.31)
Living with Child(ren)	4	3.97 (1.22)	3.64 (1.07)	100% Yes	1.75 (2.06)
Not Living with Child(ren)	544	4.11 (.85)	3.98 (0.83)	98% Yes	0.73 (1.34)
Living with Pet	184	4.18 (0.83)	3.94 (0.87)	98% Yes	0.82 (1.38)
Not Living with Pet	364	4.08 (0.86)	4.00 (0.82)	98% Yes	0.70 (1.34)
Being a Caregiver	6	4.38 (0.94)	4.24 (1.17)	100% Yes	0.67 (1.63)
Not Being a Caregiver	542	4.11 (0.85)	3.98 (0.83)	98% Yes	0.74 (1.35)

*Pre-registered analyses*. First, we examined whether household size (i.e., number of people in the household other than themselves) as a continuous measure (*M* = 2.54, range = 0 to 9 [with 77% living with 3 others or fewer], *SD* = 1.58) was associated with Time_2_ social connectedness, controlling for Time_1_ connectedness. After controlling for Time_1_ connectedness, household size did not significantly predict Time_2_ connectedness, *b* = 0.01, 95% CI = [-0.02, 0.04], *p* = .532 (see Table 3, Model 1). We then examined the association between living alone and Time_2_ social connectedness, controlling for Time_1_ connectedness. In this model, living alone (*n* = 49) was not significantly associated with Time_2_ connectedness *b* = -0.12, 95% CI = [-0.30, 0.07], *p* = .230 (see Table 3, Model 2).

**Table 3:** Results of multiple regression models (Study 1).

Model: Predictor & Dependent Variable	Adjusted R^2^	*b*(SE)	95% CI	β	*t*	*p*
*Model 1*: *Household Size & Time 2 Connectedness*	.41					
Time 1 Connectedness		0.63 (0.03)	[0.56, 0.69]	0.64	19.44	< .001
Household Size		0.01 (0.02)	[-0.02, 0.4]	0.02	0.625	.532
*Model 2*: *Living Alone & Time 2 Connectedness*	.41					
Time 1 Connectedness		0.62 (0.03)	[0.56, 0.69]	0.64	19.37	< .001
Living Alone		-0.12 (0.10)	[-0.30, 0.07]	-0.04	-1.20	.230
*Model 3*: *Living with Partner & Time 2 Connectedness*	.41					
Time 1 Connectedness		0.63 (0.03)	[0.56, 0.69]	0.64	19.64	< .001
Living with Partner		0.22 (0.08)	[0.06, 0.38]	0.09	2.65	.008
*Model 4*: *Living with Pet(s) & Time 2 Connectedness*	.41					
Time 1 Connectedness		0.63 (0.03)	[0.57, 0.69]	0.64	19.67	< .001
Living with Pet(s)		-0.12 (0.06)	[-0.24, -0.01]	-0.07	-2.10	.036
*Model 5*: *All Variables & Time 2 Connectedness*	.42					
Time 1 Connectedness		0.63 (0.03)	[0.56, 0.69]	0.64	19.651	< .001
Household Size		0.01 (0.02)	[-0.03, 0.05]	0.03	0.684	.494
Living Alone		-0.09 (0.11)	[-0.31, 0.13]	-0.03	-0.774	.439
Living with Partner		0.22 (0.08)	[0.06, 0.39]	0.09	2.656	.008
Living with Pet(s)		-0.15 (0.06)	[-0.26, -0.03]	-0.08	-2.472	.014

#### Did household composition buffer changes in social connection as a result of the COVID-19 pandemic?

**Fig 1:**
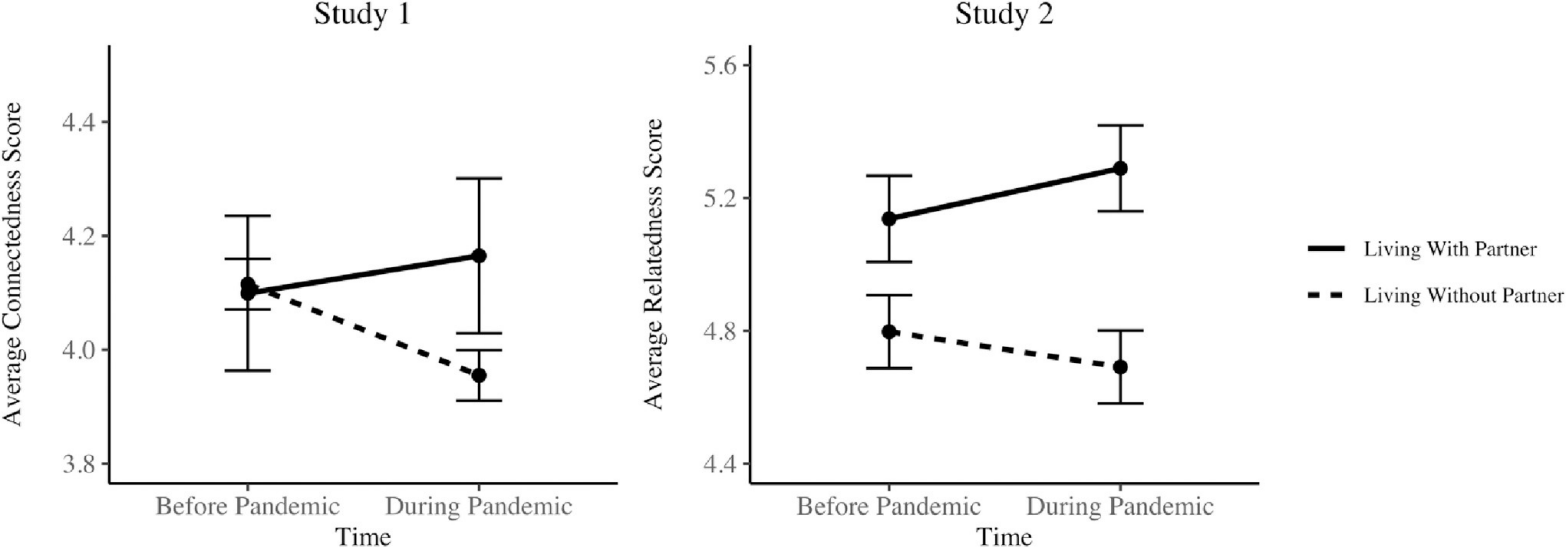
**Changes in social connection for those living with and without partners in Study 1 (left) and Study 2 (right).** *Note*. Across both Study 1 and Study 2, those living with a partner reported greater increases in social connection from before the pandemic (Time_1_) to during the pandemic (Time_2_) than those not living with a partner. Error bars represent 95% Confidence Intervals.

*Pre-registered analyses*. While household size did not appear to play a role in changes in social connectedness from before to mid-pandemic, we investigated whether features of household composition were related to Time_2_ connectedness. Controlling for Time_1_ connectedness, living with a partner (*n* = 67) predicted significantly greater social connectedness at Time_2_, *b* = 0.22, 95% CI = [0.06, 0.38], *p* = .008 (see Table 3, Model 3). See Fig 1 for an illustration of this finding. In contrast, living with a pet (*n* = 184) was associated with lower Time_2_ connectedness after controlling for Time_1_ connectedness, *b* = -0.12, 95% CI = [-0.24, -0.01], *p* = .036 (see Table 3, Model 4). Although we also pre-registered a similar analysis investigating the effects of being a caregiver on social connection, we did not conduct it, as only 6 out of 548 participants reported being a caregiver.

*Exploratory analyses*. To further investigate the relationship between household size and composition and Time_2_ social connection, we entered the household variables (household size, living alone, living with a partner, and having a pet) into a single model predicting Time_2_ social connectedness while controlling for Time_1_ connectedness. Consistent with the results of our pre-registered analyses, in this full model, living with a partner was significantly associated with higher Time_2_ social connectedness, *b* = 0.22, 95% CI = [0.06, 0.39], *p* = .008, whereas having a pet was significantly associated with lower Time_2_ social connectedness, *b* = -0.15, 95% CI = [-0.26, -0.03], *p* = .014 (see Table 3, Model 5). No other effects were significant.

#### Was the relationship between household size and changes in social connection mediated by total hours video calling with family and friends or social distancing?

*Pre-registered analyses*. It is possible that we observed no relationship between household size and shifts in social connection because individuals in smaller households may be more likely to engage in video calling or may be less likely to socially distance from non-household members. However, correlations among these variables were nonsignificant (see Table 1), precluding mediation. The pre-registered mediation analyses are presented in S1 Table.

## Study 2

Given our first study’s reliance on college students, we sought to replicate its results with a sample of adults from around the globe (U.S., U.K., and 26 other countries), who were recruited to complete our survey at two timepoints: once prior (Time_1_) and once during (Time_2_) the COVID-19 pandemic. We obtained ethics approval from the Institutional Review Board at the University of California, Riverside, and participants provided written consent to join our study. Our pre-registered stopping rules and analysis plans for Study 2 are available at [https://tinyurl.com/y8s5ssm9] on the OSF website. A portion of the data was also included in another pre-registered study [https://tinyurl.com/yc8b2n44].

## Method

### Time_1_

On February 12, 2020, participants (*N* = 396; *M*_*age*_ = 31.61, *SD*_*age*_ = 11.88; 55% Male; 80% White; 46% single/never married; 32% U.S.; 27% U.K.) completed measures of social connection, loneliness, and demographics (along with other measures that were not part of our pre-registered analysis plan). All participants were recruited from Prolific Academic^TM^, a recruitment platform demonstrated to provide quality online data [44].

### Time_2_

From April 1 to April 8, 2020, we re-recruited the same Prolific users who had completed all Time_1_ measures to participate in our Time_2_ survey. Time_2_ included the same measures assessed at Time_1_, as well as additional exploratory measures about participants’ experiences during COVID-19. Our final sample comprised 336 participants (*M*_*age*_ = 32.03, *SD*_*age*_ = 11.94; 55% Male; 80% White; 45% single/never married; 32% U.S.; 27% U.K.) who completed both Time_1_ and Time_2_ surveys and met our pre-registered inclusion criteria. A sensitivity analysis using GPower [42], assuming two-tailed α = 0.05 and 80% power, revealed the power to detect a small effect size of ƒ^2^ = .03 (R_adj_^2^ = .02) in a 2-predictor regression model and ƒ^2^ = .03 (R_adj_^2^ = .01) in a 7-predictor regression model. R_adj_^2^ is reported in the manuscript. The final dataset for Study 2 can be found on OSF at [https://tinyurl.com/yc8b2n44].

## Measures

The measures for Study 2 can be found on OSF at [https://tinyurl.com/yapg6tdt]. The same measures of (1) household size, (2) household composition (i.e., living with a partner), (3) living with pets, (4) being a caregiver, (5) social/physical distancing, and (6) hours spent video calling with family and friends were used as in Study 1.

### Social connection

Social connection in this study was assessed with two measures: (1) the 6-item relatedness subscale of the BMPN [1, 40] and (2) the 20-item UCLA Loneliness Scale [41]. The relatedness subscale asked participants to think about the past week and rate agreement with statements such as, “I felt close and connected with other people who are important to me” (1 = *strongly disagree*, 7 = *strongly agree*). Relatedness scores were highly reliable at both Time_1_ (*α =* .76) and Time_2_ (*α =* .77). The UCLA Loneliness Scale prompted participants to respond to statements based on how they feel in general (e.g., “People are around me but not with me”; 1 = *never*, 4 = *often*). Loneliness scores were highly reliable at both Time_1_ (*α =* .88) and Time_2_ (*α* = .88).

### Hours spent working outside the home

Participants were additionally asked “how many hours per week do you work outside the home?” with answer choices ranging from “0” to “40+ hours.”

### Additional exclusion criteria

As pre-registered, to screen out inattentive participants, we planned to exclude those who provided the same answer 15 times in a row on the 20-item UCLA Loneliness Scale. We also pre-registered to exclude those who were missing more than 1 item on the 6-item BMPN relatedness subscale and missing more than 2 items on the UCLA Loneliness Scale. However, we did not have any instances of inattentiveness or missing data.

### Study 2 results

The R code used for the analyses in Study 2 can be found on OSF at [https://tinyurl.com/y7nhpx7h]. Correlations among variables in Study 2 can be found in Table 4.

**Table 4:** Correlations among variables (Study 2).

	Household Size	Living Alone	Living with Partner	Living with Child(ren)	Living with Pet	Being a Caregiver	Hours Video Calling	Social Distancing	T1 Relatedness	T2 Relatedness	T1 Loneliness	T2 Loneliness
Household Size	1											
Living Alone	-.62***	1										
Living with Partner	.11*	-.34***	1									
Living with Child(ren)	.29***	-.24***	.58***	1								
Living with Pet	.13*	-.17**	.20***	.09	1							
Being a Caregiver	.20***	-.21***	.47***	.65***	.08	1						
Hours Video Calling	.11*	-.08	-.07	-.03	.11	.00	1					
Social Distancing	.06	-.04	.01	.00	-.08	-.02	-.29***	1				
T1 Relatedness	-.01	.02	.15**	.09	.14**	.09	.00	-.03	1			
T2 Relatedness	-.01	-.06	.25***	.14*	.15**	.13*	.00	-.02	.50***	1		
T1 Loneliness	-.10	.15**	-.22***	-.16**	-.13*	-.13*	-.08	.01	-.67***	-.47***	1	
T2 Loneliness	-.11	.12*	-.20***	-.12*	-.14*	-.07	-.04	.04	-.58***	-.63***	.80***	1

Note.

*** = p < .001.

** = p < .01.

* = p < .05.

**Did household size buffer changes in social connection as a result of the COVID-19 pandemic?.** As reported by Folk et al. (2020), our sample showed no changes in relatedness and small but significant improvements in loneliness from before to after the pandemic. Additionally, 93% of participants reported that they were social distancing (see Table 5).

**Table 5:** Means and standard deviations for household size and composition (Study 2).

Household Variable	Sample Size	Time 1 Relatedness	Time 2 Relatedness	Time 1 Loneliness	Time 2 Loneliness	Social Distancing	Six Feet
Household Size	336	4.92 (1.09)	4.91 (1.14)	2.20 (0.51)	2.16 (0.49)	93% Yes	1.12 (1.75)
Living Alone	55	4.98 (1.02)	4.75 (1.10)	2.37 (0.52)	2.29 (0.50)	91% Yes	1.24 (2.01)
Not Living Alone	281	4.91 (1.10)	4.94 (1.15)	2.17 (0.50)	2.13 (0.48)	94% Yes	1.09 (1.70)
Living with Partner	124	5.14 (1.04)	5.29 (1.05)	2.06 (0.50)	2.03 (0.46)	94% Yes	1.14 (1.71)
Not Living with Partner	212	4.80 (1.09)	4.69 (1.14)	2.29 (0.50)	2.23 (0.49)	93% Yes	1.10 (1.78)
Living with Child(ren)	74	5.11 (1.08)	5.20 (1.06)	2.05 (0.49)	2.05 (0.47)	93% Yes	1.28 (1.69)
Not Living with Child(ren)	262	4.87 (1.08)	4.83 (1.15)	2.25 (0.51)	2.19 (0.49)	93% Yes	1.07 (1.77)
Living with Pet	168	5.08 (1.10)	5.08 (1.20)	2.14 (0.51)	2.09 (0.49)	91% Yes	1.07 (1.69)
Not Living with Pet	168	4.77 (1.05)	4.74 (1.06)	2.27 (0.51)	2.22 (0.48)	95% Yes	1.17 (1.82)
Being a Caregiver	63	5.13 (0.96)	5.22 (1.07)	2.06 (0.47)	2.09 (0.47)	92% Yes	1.41 (1.71)
Not Being a Caregiver	273	4.88 (1.11)	4.84 (1.15)	2.24 (0.52)	2.17 (0.49)	93% Yes	1.05 (1.76)

*Pre-registered analyses*. We first examined whether a continuous measure of household size (*M* = 2.38, range = 0 to 5 [with 88% living with 2 others or fewer], *SD* = 0.98) was associated with our two measures of Time_2_ social connection (relatedness and loneliness), after controlling for Time_1_ social connection. Similar to Study 1, after controlling for Time_1_ social connection, household size did not predict Time_2_ social connection for relatedness, *b* = -.003, 95% CI = [-0.11, 0.10], *p* = .954 (see Table 6, Model 1) or loneliness, *b* = -0.01, 95% CI [-0.04, 0.02], *p* = .456 (see Table 6, Model 2). Similarly, living alone (*n* = 55) compared to not living alone (*n* = 281) was not reliably associated with Time_2_ social connection for relatedness, *b* = -.23, 95% CI [-0.51, 0.06], *p* = .119 (see Table 6, Model 3) or loneliness, *b* = .004, 95% CI [ -0.08, 0.09], *p* = .925 (see Table 6, Model 4) after controlling for Time_1_ social connection.

**Table 6:** Results of multiple regression models (Study 2).

Model: Predictor & Dependent Variable	Adjusted R^2^	*b*(SE)	95% CI	β	*t*	*p*
*Model 1*: *Household Size & Time 2 Relatedness*	.25					
Time 1 Relatedness		.53 (.05)	[0.43, 0.63]	.50	10.611	< .001
Household Size		-.003 (.05)	[-0.11, 0.10]	-.003	-0.058	.954
*Model 2*: *Household Size & Time 2 Loneliness*	.65					
Time 1 Loneliness		.77 (.03)	[0.70, 0.83]	.80	24.553	< .001
Household Size		-.01 (.02)	[-0.04, 0.02]	-.02	-0.747	.456
*Model 3*: *Living Alone & Time 2 Relatedness*	.25					
Time 1 Relatedness		.53 (.05)	[0.43, 0.63]	.50	10.685	< .001
Living Alone		-.23 (.15)	[-0.51, 0.06]	-.07	-1.562	.119
*Model 4*: *Living Alone & Time 2 Loneliness*	.65					
Time 1 Loneliness		.77 (.03)	[0.71, 0.83]	.80	24.464	< .001
Living Alone		.00 (.05)	[-0.08, 0.13]	.00	0.094	.925
*Model 5*: *Living with Partner & Time 2 Relatedness*	.28					
Time 1 Relatedness		.50 (.05)	[0.40, 0.60]	.48	10.139	< .001
Living with Partner		.43 (.11)	[0.21, 0.65]	.18	3.864	< .001
*Model 6*: *Living with Partner & Time 2 Loneliness*	.65					
Time 1 Loneliness		.76 (.03)	[0.70, 0.83]	.80	24.006	< .001
Living with Partner		-.03 (.03)	[-0.09, 0.04]	-.03	-0.801	.424
*Model 7*: *Living with Child(ren) & Time 2 Relatedness*	.26					
Time 1 Relatedness		.52 (.05)	[0.42, 0.62]	.49	10.452	< .001
Living with Child(ren)		.25 (.13)	[-0.004, 0.51]	.09	1.94	.053
*Model 8*: *Living with Child(ren) & Time 2 Loneliness*	.65					
Time 1 Loneliness		.77 (.03)	[0.71, 0.83]	.81	24.465	< .001
Living with Child(ren)		.01 (.04)	[-0.07, 0.08]	.01	0.194	.846
*Model 9*: *Living with Pet(s) & Time 2 Relatedness*	.25					
Time 1 Relatedness		.52 (.05)	[0.42, 0.62]	.49	10.302	< .001
Living with Pet(s)		.18 (.11)	[-0.03, 0.40]	.08	1.687	.093
*Model 10*: *Living with Pet(s) & Time 2 Loneliness*	.65					
Time 1 Loneliness		.77 (.03)	[0.70, 0.83]	.80	24.432	< .001
Living with Pet(s)		-.03 (.04)	[-0.09, 0.04]	-.03	-0.810	.419
*Model 11*: *Being a Caregiver & Time 2 Relatedness*	.26					
Time 1 Relatedness		.52 (.05)	[0.42, 0.62]	.50	10.458	< .001
Being a Caregiver		.25 (.14)	[-0.02, 0.52]	.08	1.793	.074
*Model 12*: *Being a Caregiver & Time 2 Loneliness*	.65					
Time 1 Loneliness		.77 (.03)	[0.71, 0.84]	.81	25.742	< .001
Being a Caregiver		.05 (.04)	[-0.03, 0.13]	.04	1.226	.221

#### Did household composition buffer changes in social connection as a result of the COVID-19 pandemic?

*Pre-registered analyses*. Next, we tested whether aspects of household composition were associated with Time_2_ social connection, controlling for Time_1_ levels of social connection, for our two measures of social connection (loneliness and relatedness). None of the household composition variables were significantly associated with Time_2_ loneliness, when controlling for Time_1_ loneliness (see Table 6). However, consistent with Study 1, living with a partner (*n* = 124) was associated with greater Time_2_ relatedness after controlling for Time_1_ relatedness, *b* = .43, 95% CI [0.21, 0.65], *p* < .001 (see Table 6, Model 5; see Fig 1 for an illustration of this finding). We repeated the same analysis with each of the other household composition variables. Living with children was linked to marginally greater Time_2_ relatedness after controlling for Time_1_ relatedness, (*n* = 74; *b* = .25, 95% CI [-0.004, 0.51], *p* = .053 (see Table 6, Model 7). Finally, living with pets (*n* = 168; *b* = .18, 95% CI [-0.03, 0.40], *p* = .093 (see Table 6, Model 9) and being a caregiver (*n* = 63; *b* = .25, 95% CI [-0.02, 0.52], *p* = .074 (see Table 6, Model 11) showed similar marginal positive effects.

*Exploratory analyses*. As in Study 1, we examined which aspects of household size and composition—when tested in a single model—best predicted Time_2_ social connection after controlling for Time_1_ social connection. None of the household size and composition variables were significantly associated with Time_2_ loneliness, when controlling for Time_1_ loneliness (see Table 7, Model 14). However, when we examined the same variables (household size, living alone, living with a partner, living with a child, living with a pet, and being a caregiver) in a single model predicting Time_2_ relatedness, controlling for Time_1_ relatedness, living with a partner was the only factor that buffered changes in social connection, *b* = .38, 95% CI. [0.09, 0.67], *p* = .012 (see Table 7, Model 13). This finding was consistent with Study 1.

**Table 7:** Results of exploratory multiple regression models (Study 2).

Model: Predictor & Dependent Variable	Adjusted R^2^	*b*(SE)	95% CI	β	*t*	*p*
*Model 13*: *Household Size/Composition & Relatedness*	.27					
Time 1 Relatedness		.49 (.05)	[0.40, 0.59]	.47	9.873	< .001
Household Size		-.06 (.07)	[-0.21, 0.08]	-.05	-0.863	.389
Living Alone		-.13 (.20)	[-0.52, 0.26]	-.04	-0.657	.512
Living with Partner		.38 (.15)	[0.09, 0.67]	.16	2.540	.012
Living with Child		-.02 (.19)	[-0.39, 0.36]	-.01	-0.085	.932
Living with Pet		.12 (.11)	[-0.10, 0.33]	.05	1.052	.294
Being a Caregiver		.04 (.18)	[-0.32, 0.39]	.01	0.215	.830
*Model 14*: *Household Size/Composition & Loneliness*	.65					
Time 1 Loneliness		.76 (.03)	[0.70, 0.83]	.81	23.789	< .001
Household Size		-.03 (.02)	[-0.07, 0.02]	-.06	-1.280	.202
Living Alone		-.05 (.06)	[-0.17, 0.06]	-.03	-0.904	.367
Living with Partner		-.07 (.04)	[-0.16, 0.02]	-.07	-1.530	.127
Living with Child		.01 (.06)	[-0.10, 0.12]	.02	0.142	.887
Living with Pet		-.02 (.03)	[-0.08, 0.05]	-.03	-0.591	.555
Being a Caregiver		.09 (.05)	[-0.02, 0.19]	.07	1.633	.104

#### Did working outside of the home moderate the effects of household size and composition on changes in social connection?

*Pre-registered analyses*. We expected that household size and household composition might matter less for social connection for individuals who worked outside the home. However, we did not find that hours working outside the home moderated the relationship between household size (continuous and living alone) or composition (living with a partner, living with children, living with a pet, being a caregiver) and changes in relatedness or loneliness (see S2 Table).

#### Was the relationship between household size and changes in social connection mediated by total hours video calling with family and friends or social distancing?

*Pre-registered analyses*. No significant correlations emerged between our outcome variable (relatedness, loneliness) and 1) our predictor variable (household size) and 2) our mediator variables (hours video calling, social distancing; see Table 4 for correlations). Thus, parallel to Study 1, the number of hours spent video calling with family and friends or social distancing did not mediate the relationship between household size (continuous and living alone) and changes in relatedness or loneliness (see S3 Table).

## Discussion

Across two pre-registered studies that followed the same participants from before the COVID-19 pandemic into its early stages, we found that living with a partner was the strongest predictor of shifts in social connection across time. This finding replicated across two different samples—a sample of undergraduates at a Canadian university and a sample of adults from mostly the U.S. and the U.K. Both of our studies revealed robust positive regression coefficients indicating that people living with a partner were more likely to improve in social connection after social distancing guidelines were in place than those not living with a partner. This finding is consistent with past research demonstrating that being in a relationship is one of the strongest predictors of connection and well-being [11, 45], in part because happier people are more likely to find partners [46, 47]. Additionally, during times of worry and uncertainty, partners have been found to be more valuable for coping than other types of household members [26]. Moreover, recent research has shown that, on average, romantic relationships have not deteriorated over the course of the pandemic; indeed, people are relatively more willing to forgive their partners during COVID-19 [48]. In light of this evidence, it is not surprising that partners showed the strongest effect, especially during a pandemic.

Contrary to our pre-registered hypotheses, changes in loneliness were not predicted by any other aspects of household composition. Furthermore, we found only nonsignificant trends for the impact of household size, including living alone, on social connection during COVID-19, perhaps because both our studies included small samples of those living in large households and households of one. It is important to keep in mind that the pandemic has forced people to spend unusually large amounts of time confined to home. Given that interpersonal interactions must be positive to contribute to one’s overall sense of connectedness [10], those who live in larger households—relative to those who live alone or in smaller households—may have had more interactions that were negative (e.g., due to bickering or lack of privacy and alone time) and, as a result, failed to experience benefits in terms of social connection. Moreover, our studies measured experiences fairly early in the pandemic (April 2020); thus, as people continue to distance over long periods of time, their feelings of social connection may suffer. Going beyond household size and structure, future studies should examine the effects of relationship quality on social connection over time.

When examining how other features of household composition were associated with shifts in social connection during the pandemic, we obtained mixed findings regarding living with pets and null findings for all other household variables. However, because households are multifaceted, larger sample sizes will be needed to fully dissect the household composition findings, as well as to reveal interactions (such as with household size, gender, or country of residence). For example, studies with larger sample sizes may uncover differences in connection between those in households of four (with a partner and two children) versus households of five (with a partner and three children), and so on. Importantly, future investigators may wish to further unpack the role of household dynamics, as some households include unhealthy relationships that may be exacerbated by social distancing measures and others include housemates that minimally interact. As such, the quality and frequency of interaction among household members—perhaps with experience sampling or daily diary measures—is an important factor to explore in future work.

### Implications and conclusions

Directed by social distancing interventions in the spring of 2020, millions of people were no longer commuting to work, attending school, or leaving their homes to spend time with friends and family. These extraordinary conditions likely led people to rely more on their household members to fulfill their needs for closeness, belonging, and connection [10]. The results from our two studies revealed that living with a partner—but not how many people or who else one lives with—appeared to confer unique benefits during these uncertain and unprecedented times. Indeed, demonstrating its robustness, this finding replicated across our two studies, despite weak and opposite correlations between household size and living with a partner (*r* = -.06 in Study 1 and .11 in Study 2).

In light of these results, policy makers might consider developing guidelines for social/physical distancing that protect people’s physical health while ensuring they retain a sense of closeness and connection by spending time in close proximity with partners, even outside their households. Some areas in the world, such as New Zealand, have implemented a strategy known as the “social bubble,” which is the easing of social distancing to allow close contact with another household [49]. Such approaches might be especially helpful for individuals who have been unintentionally and disproportionally socially isolated by social distancing measures, such as those who are cut-off, separated from their partners, or generally struggling with staying at home. However, social bubbles pose a risk of increased infection rates [49]. Hence, just as safe sex education aims to reduce the rate of sexually transmitted diseases and unintended pregnancy, education on safe social distancing (or social bubbling) strategies might guide individuals across the globe how to connect with others safely while simultaneously curtailing COVID-19 rates. In sum, recommendations that reduce the risk of transmission while prioritizing social connection can ensure that people’s physical and psychological health are optimally balanced.

## Supporting information

S1 Table Results of mediation analyses (Study 1). (DOCX)

S2 Table Results of moderation analyses for hours spent working (Study 2). (DOCX)

S3 Table Results of mediation analyses (Study 2). (DOCX)
