# Supplementary material for: Changes in social connection during COVID-19 social distancing: It’s not (household) size that matters, it’s who you’re with
Source: PLoS One. 2021 Jan 20;16(1):e0245009. doi: 10.1371/journal.pone.0245009 (PMC7817035; doi:10.1371/journal.pone.0245009)
Supplement: S1 Table — (DOCX) [file pone.0245009.s001.docx]

**S1 Table. Results of Mediation Analyses (Study 1).**

| Model | Label | Estimate | SE | 95% CI | Z | *p* |
| --- | --- | --- | --- | --- | --- | --- |
| *Mediation 1* |  |  |  |  |  |  |
| Household Size 🡪 Time_2_ Social Connectedness | c | 0.01 | 0.02 | [-0.02, 0.04] | 0.662 | .508 |
| Household Size 🡪 Time_2_ Social Connectedness | c' | 0.01 | 0.02 | [-0.02. 0.04] | 0.695 | .487 |
| Indirect Effect of Video Calling with Family/Friends | a x b | -0.001 | 0.002 | [-0.004, 0.003] | -0.426 | .670 |
| *Mediation 2* |  |  |  |  |  |  |
| Living Alone 🡪 Time_2_ Social Connectedness | c | -0.12 | 0.09 | [-0.29, 0.06] | -1.317 | .188 |
| Living Alone 🡪 Time_2_ Social Connectedness | c' | -0.12 | 0.09 | [-0.30, 0.06] | -1.340 | .180 |
| Indirect Effect of Video Calling with Family/Friends | a x b | 0.01 | 0.01 | [-0.01, 0.03] | 0.633 | .527 |
| *Mediation 3* |  |  |  |  |  |  |
| Household Size 🡪 Time_2_ Social Connectedness | c | 0.01 | 0.02 | [-0.02, 0.04] | 0.662 | .508 |
| Household Size 🡪 Time_2_ Social Connectedness | c' | 0.01 | 0.02 | [-0.02, 0.04] | 0.645 | .519 |
| Indirect Effect of Social Distancing (Yes/No) | a x b | 0.000 | 0.001 | [-0.002, 0.002] | 0.249 | .803 |
| *Mediation 4* |  |  |  |  |  |  |
| Living Alone 🡪 Time_2_ Social Connectedness | c | -0.12 | 0.09 | [-0.29, 0.06] | -1.317 | .188 |
| Living Alone 🡪 Time_2_ Social Connectedness | c' | -0.11 | 0.09 | [-0.29, 0.06] | -1.292 | .196 |
| Indirect Effect of Social Distancing (Yes/No) | a x b | -0.003 | 0.01 | [-0.02, 0.03] | -0.223 | .823 |
| *Mediation 5* |  |  |  |  |  |  |
| Household Size 🡪 Time_2_ Social Connectedness | c | 0.01 | 0.02 | [-0.02, 0.04] | 0.662 | .508 |
| Household Size 🡪 Time_2_ Social Connectedness | c' | 0.01 | 0.02 | [-0.02, 0.05] | 0.740 | .459 |
| Indirect Effect of Social Distancing (People within Six Feet) | a x b | -0.001 | 0.002 | [-0.006, 0.001] | -0.792 | .428 |
| *Mediation 6* |  |  |  |  |  |  |
| Living Alone 🡪 Time_2_ Social Connectedness | c | -0.12 | 0.09 | [-0.29, 0.06] | -1.317 | .188 |
| Living Alone 🡪 Time_2_ Social Connectedness | c' | -0.11 | 0.09 | [-0.29, 0.06] | -1.291 | .197 |
| Indirect Effect of Social Distancing (People within Six Feet) | a x b | -0.002 | 0.01 | [-0.02, 0.01] | -0.374 | .708 |

*Note.* Each mediation analysis controlled for Time 1 social connection. Path coefficients and confidence intervals were estimated with 5,000 bootstrapped samples.
