# Supplementary material for: Changes in social connection during COVID-19 social distancing: It’s not (household) size that matters, it’s who you’re with
Source: PLoS One. 2021 Jan 20;16(1):e0245009. doi: 10.1371/journal.pone.0245009 (PMC7817035; doi:10.1371/journal.pone.0245009)
Supplement: S2 Table — (DOCX) [file pone.0245009.s002.docx]

**S2 Table. Results of Moderation Analyses for Hours Spent Working (Study 2).**

| Moderation: Predictor & Dependent Variable | Adjusted R^2^ | *b*(SE) | 95% CI | β | *t* | *p* |  |
| --- | --- | --- | --- | --- | --- | --- | --- |
| *Moderation 1: Household Size & Time 2 Relatedness* | .24 |  |  |  |  |  |  |
| Time 1 Relatedness |  | .53 (.05) | [0.43, 0.63] | .50 | 10.568 | < .001 |  |
| Household Size |  | -.002 (.07) | [-0.14, 0.14] | -.002 | -0.024 | .973 |  |
| Hours Spent Working |  | .005 (.05) | [0.10, 0.11] | .01 | 0.092 | .926 |  |
| Household Size x Hours Spent Working |  | .000 (.02) | [-0.04, 0.04] | -.002 | -0.013 | .990 |  |
| *Moderation 2: Household Size & Time 2 Loneliness* | .65 |  |  |  |  |  |  |
| Time 1 Loneliness |  | .76 (.03) | [0.70, 0.83] | .80 | 24.344 | < .001 |  |
| Household Size |  | -.01 (.02) | [-0.06, 0.03] | -.03 | -0.652 | .515 |  |
| Hours Spent Working |  | -.01 (.02) | [-0.04, 0.02] | -.04 | -0.475 | .635 |  |
| Household Size x Hours Spent Working |  | .000 (.01) | [-0.01, 0.01] | -.001 | .008 | .993 |  |
| *Moderation 3: Living Alone & Time 2 Relatedness* | .25 |  |  |  |  |  |  |
| Time 1 Relatedness |  | .53 (.05) | [0.43, 0.63] | .50 | 10.627 | < .001 |  |
| Living Alone |  | -.21 (.20) | [-0.60, 0.18] | -.07 | -1.069 | .286 |  |
| Hours Spent Working |  | .01 (.02) | [-0.04, 0.06] | .02 | 0.397 | .692 |  |
| Living Alone x Hours Spent Working |  | -.01 (.05) | [-0.11, 0.09] | -.01 | -0.170 | .865 |  |
| *Moderation 4: Living Alone & Time 2 Loneliness* | .65 |  |  |  |  |  |  |
| Time 1 Loneliness |  | .76 (.03) | [0.70, 0.83] | .80 | 24.243 | < .001 |  |
| Living Alone |  | -.03 (.06) | [-0.14, 0.09] | -.02 | -0.443 | .658 |  |
| Hours Spent Working |  | -.01 (.01) | [-0.02, 0.00] | -.05 | -1.438 | .151 |  |
| Living Alone x Hours Spent Working |  | .01 (.01) | [-0.02, 0.04] | .04 | 0.919 | .359 |  |
| *Moderation 5: Living with Partner & Time 2 Relatedness* | .28 |  |  |  |  |  |  |
| Time 1 Relatedness |  | .50 (.05) | [0.40, 0.60] | .47 | 10.119 | < .001 |  |
| Living with Partner |  | .32 (.15) | [0.04, 0.61] | .14 | 2.219 | .027 |  |
| Hours Spent Working |  | -.02 (.03) | [-0.08, 0.03] | -.06 | -0.883 | .378 |  |
| Living with Partner x Hours Spent Working |  | .05 (.04) | [-0.03, 0.13] | .09 | 1.142 | .254 |  |
| *Moderation 6: Living with Partner & Time 2 Loneliness* | .65 |  |  |  |  |  |  |
| Time 1 Loneliness |  | .76 (.03) | [0.70, 0.82] | .79 | 23.897 | < .001 |  |
| Living with Partner |  | -.04 (.04) | [-0.12, 0.05] | -.04 | -0.851 | .395 |  |
| Hours Spent Working |  | -.01 (.01) | [-0.03, 0.01] | -.05 | -1.094 | .275 |  |
| Living with Partner x Hours Spent Working |  | .01 (.01) | [-0.02, 0.03] | -.03 | 0.472 | .637 |  |
| *Moderation 7: Living with Child(ren) & Time 2 Relatedness* | .25 |  |  |  |  |  |  |
| Time 1 Relatedness |  | .52 (.05) | [0.42, 0.62] | .49 | 10.421 | < .001 |  |
| Living with Child(ren) |  | .27 (.18) | [-0.08, 0.62] | .10 | 1.541 | .124 |  |
| Hours Spent Working |  | .00 (.03) | [-0.05, 0.05] | .002 | 0.038 | .970 |  |
| Living with Child(ren) x Hours Spent Working |  | -.01 (.04) | [-0.09, 0.08] | -.01 | -0.160 | .873 |  |
| *Moderation 8: Living with Child(ren) & Time 2 Loneliness* | .65 |  |  |  |  |  |  |
| Time 1 Loneliness |  | .77 (.03) | [0.71, 0.83] | .81 | 24.364 | < .001 |  |
| Living with Child(ren) |  | -.01 (.05) | [-0.12, 0.09] | -.01 | -0.239 | .811 |  |
| Hours Spent Working |  | -.01 (.01) | [-0.02, 0.00] | -.05 | -1.381 | .168 |  |
| Living with Child(ren) x Hours Spent Working |  | .01 (.01) | [-0.02, 0.04] | .04 | 0.755 | .451 |  |
| *Moderation 9: Living with Pet & Time 2 Relatedness* | .25 |  |  |  |  |  |  |
| Time 1 Relatedness |  | .52 (.05) | [0.42, 0.62] | .49 | 10.268 | < .001 |  |
| Living with Pet |  | .15 (.14) | [-0.14, 0.43] | .06 | 1.020 | .308 |  |
| Hours Spent Working |  | -.01 (.03) | [-0.07, 0.05] | -.01 | -0.206 | .837 |  |
| Living with Pet x Hours Spent Working |  | .02 (.04) | [-0.07, 0.10] | .03 | 0.389 | .698 |  |
| *Moderation 10: Living with Pet & Time 2 Loneliness* | .65 |  |  |  |  |  |  |
| Time 1 Loneliness |  | .76 (.03) | [0.70, 0.82] | .80 | 24.280 | < .001 |  |
| Living with Pet |  | -.03 (.04) | [-0.11, 0.05] | -.03 | -0.709 | .479 |  |
| Hours Spent Working |  | -.01 (.01) | [-0.03, 0.01] | -.04 | -0.867 | .386 |  |
| Living with Pet x Hours Spent Working |  | .002 (.01) | [-0.02, 0.03] | .01 | 0.195 | .846 |  |
| *Moderation 11: Being a Caregiver & Time 2 Relatedness* | .25 |  |  |  |  |  |  |
| Time 1 Relatedness |  | .52 (.05) | [0.42, 0.62] | .49 | 10.400 | < .001 |  |
| Being a Caregiver |  | .32 (.19) | [-0.05, 0.68] | .11 | 1.711 | .088 |  |
| Hours Spent Working |  | .01 (.02) | [-0.04, 0.05] | .02 | 0.333 | .739 |  |
| Being a Caregiver x Hours Spent Working |  | -.03 (.05) | [-0.12, 0.07] | -.04 | -0.574 | .567 |  |
| *Moderation 12: Being a Caregiver & Time 2 Loneliness* | .65 |  |  |  |  |  |  |
| Time 1 Loneliness |  | .77 (.03) | [0.71, 0.83] | .81 | 24.629 | < .001 |  |
| Being a Caregiver |  | .03 (.05) | [-0.08, 0.13] | .02 | 0.499 | .618 |  |
| Hours Spent Working |  | -.01 (.01) | [-0.02, 0.00] | -.05 | -1.436 | .152 |  |
| Being a Caregiver x Hours Spent Working |  | .01 (.01) | [-0.02, 0.04] | .04 | 0.750 | .454 |  |

*Note.* Hours spent working was measuring all hours spent working outside of the home.
