# Supplementary material for: Changes in social connection during COVID-19 social distancing: It’s not (household) size that matters, it’s who you’re with
Source: PLoS One. 2021 Jan 20;16(1):e0245009. doi: 10.1371/journal.pone.0245009 (PMC7817035; doi:10.1371/journal.pone.0245009)
Supplement: S3 Table — (DOCX) [file pone.0245009.s003.docx]

**S3 Table. Results of Mediation Analyses (Study 2).**

| Model | Label | Estimate | SE | Z | *p* | 95% CI |
| --- | --- | --- | --- | --- | --- | --- |
| *Mediation 1* |  |  |  |  |  |  |
| Household Size 🡪 Time 2 Relatedness | c | -.003 | .062 | -.052 | .959 | [-0.13, 0.11] |
| Household Size 🡪 Time 2 Relatedness | c' | -.003 | .062 | -.051 | .959 | [-0.13, 0.11] |
| Indirect Effect of VC Family/Friends | a x b | .000 | .006 | .001 | .999 | [-0.01, 0.01] |
| *Mediation 2* |  |  |  |  |  |  |
| Living Alone 🡪 Time 2 Relatedness | c | -.227 | .152 | -1.496 | .135 | [-0.52, 0.07] |
| Living Alone 🡪 Time 2 Relatedness | c' | -.229 | .152 | -1.500 | .134 | [-0.52, 0.07] |
| Indirect Effect of VC Family/Friends | a x b | .001 | .011 | .134 | .894 | [-0.02, 0.03] |
| *Mediation 3* |  |  |  |  |  |  |
| Household Size 🡪 Time 2 Loneliness | c | -.012 | .015 | -.796 | .426 | [-0.04, 0.02] |
| Household Size 🡪 Time 2 Loneliness | c' | -.013 | .015 | -.887 | .375 | [-0.04, 0.02] |
| Indirect Effect of VC Family/Friends | a x b | .001 | .002 | .698 | .485 | [-0.00, 0.01] |
| *Mediation 4* |  |  |  |  |  |  |
| Living Alone 🡪 Time 2 Loneliness | c | .004 | .048 | .086 | .931 | [-0.09, 0.09] |
| Living Alone 🡪 Time 2 Loneliness | c' | .006 | .048 | .131 | .896 | [-0.09, 0.10] |
| Indirect Effect of VC Family/Friends | a x b | -.002 | .004 | -.561 | .575 | [-0.01, 0.01] |
| *Mediation 5* |  |  |  |  |  |  |
| Household Size 🡪 Time 2 Relatedness | c | -.003 | .062 | -.051 | .959 | [-0.13, 0.11] |
| Household Size 🡪 Time 2 Relatedness | c' | -.003 | .062 | -.045 | .964 | [-0.13, 0.11] |
| Indirect Effect of Social Distancing | a x b | .000 | .004 | -.093 | .926 | [-0.01, 0.01] |
| *Mediation 6* |  |  |  |  |  |  |
| Living Alone 🡪 Time 2 Relatedness | c | -.227 | .152 | -1.496 | .135 | [-0.52, 0.07] |
| Living Alone 🡪 Time 2 Relatedness | c' | -.228 | .153 | -1.490 | .136 | [-0.53, 0.07] |
| Indirect Effect of Social Distancing | a x b | .001 | .010 | .101 | .919 | [-0.02, 0.03] |
| *Mediation 7* |  |  |  |  |  |  |
| Household Size 🡪 Time 2 Loneliness | c | -.012 | .015 | -.796 | .426 | [-0.04, 0.02] |
| Household Size 🡪 Time 2 Loneliness | c' | -.013 | .015 | -.858 | .391 | [-0.04, 0.02] |
| Indirect Effect of Social Distancing | a x b | .001 | .002 | .597 | .550 | [-0.00, 0.01] |
| *Mediation 8* |  |  |  |  |  |  |
| Living Alone 🡪 Time 2 Loneliness | c | .004 | .048 | .086 | .931 | [-0.09, 0.09] |
| Living Alone 🡪 Time 2 Loneliness | c' | .006 | .047 | .124 | .902 | [-0.09, 0.10] |
| Indirect Effect of Social Distancing | a x b | -.002 | .004 | -.407 | .684 | [-0.01, 0.00] |
| *Mediation 9* |  |  |  |  |  |  |
| Household Size 🡪 Time 2 Relatedness | c | -.003 | .062 | -.051 | .959 | [-0.13, 0.11] |
| Household Size 🡪 Time 2 Relatedness | c' | -.002 | .062 | -.034 | .973 | [-0.13, 0.12] |
| Indirect Effect of Six Feet | a x b | -.001 | .004 | -.282 | .778 | [-0.01, 0.01] |
| *Mediation 10* |  |  |  |  |  |  |
| Living Alone 🡪 Time 2 Relatedness | c | -.227 | .152 | -1.496 | .135 | [-0.52, 0.07] |
| Living Alone 🡪 Time 2 Relatedness | c' | -.230 | .152 | -1.515 | .130 | [-0.53, 0.07] |
| Indirect Effect of Six Feet | a x b | .003 | .011 | .278 | .781 | [-0.02, 0.03] |
| *Mediation 11* |  |  |  |  |  |  |
| Household Size 🡪 Time 2 Loneliness | c | -.012 | .015 | -.787 | .432 | [-0.04, 0.02] |
| Household Size 🡪 Time 2 Loneliness | c' | -.013 | .016 | -.809 | .418 | [-0.04, 0.02] |
| Indirect Effect of Six Feet | a x b | .000 | .001 | .365 | .715 | [0.00, 0.00] |
| *Mediation 12* |  |  |  |  |  |  |
| Living Alone 🡪 Time 2 Loneliness | c | .004 | .048 | .086 | .931 | [-0.09, 0.10] |
| Living Alone 🡪 Time 2 Loneliness | c' | .005 | .048 | .110 | .912 | [-0.09, 0.10] |
| Indirect Effect of Six Feet | a x b | -.001 | .003 | -.339 | .735 | [-0.01, 0.01] |

*Note.* Each mediation analysis controlled for Time 1 social connection. Path coefficients and confidence intervals were estimated with 5,000 bootstrapped samples.
